# ClusPro PeptiDock: efficient global docking of peptide recognition motifs using FFT

**DOI:** 10.1093/bioinformatics/btx216

**Published:** 2017-04-18

**Authors:** Kathryn A Porter, Bing Xia, Dmitri Beglov, Tanggis Bohnuud, Nawsad Alam, Ora Schueler-Furman, Dima Kozakov

**Affiliations:** 1 Department of Biomedical Engineering, Boston University, Boston, MA, USA; 2 Department of Microbiology, Hebrew University, Jerusalem, Israel; 3 Department of Applied Mathematics and Statistics, Stony Brook University, Stony Brook, NY, USA; 4 Laufer Center for Physical and Quantitative Biology, Stony Brook University, Stony Brook, NY, USA

## Abstract

**Summary:**

We present an approach for the efficient docking of peptide motifs to their free receptor structures. Using a motif based search, we can retrieve structural fragments from the Protein Data Bank (PDB) that are very similar to the peptide’s final, bound conformation. We use a Fast Fourier Transform (FFT) based docking method to quickly perform global rigid body docking of these fragments to the receptor. According to CAPRI peptide docking criteria, an acceptable conformation can often be found among the top-ranking predictions.

**Availability and Implementation:**

The method is available as part of the protein-protein docking server ClusPro at https://peptidock.cluspro.org/nousername.php.

**Supplementary information:**

Supplementary data are available at *Bioinformatics* online.

## 1 Introduction

Fast Fourier Transform (FFT) based sampling approaches have been shown to be very effective for modeling protein–protein interactions. If we assume that proteins undergo minimal conformational changes upon binding, we can exhaustively sample mutual protein orientations using a molecular mechanics like energy function in an efficient manner (Chen *et al.*, 2003; Kozakov *et al.*, 2006; Tovchigrechko and Vakser, 2006; Viswanath *et al.*, 2014). This assumption has been adequate in the Critical Assessment of PRediction of Interactions (CAPRI), a community wide docking experiment (Lensink *et al.*, 2016) where FFT based approaches have consistently been among the top performers (Lensink and Wodak, 2013). The low cost of such techniques that do not require prior knowledge of the binding site makes them well suited for implementation as an automated server. In particular, our ClusPro server (Kozakov *et al.*, 2013) is consistently the most accurate server according to CAPRI evaluations, and performs comparably to the best human groups (Lensink *et al.*, 2016). ClusPro is heavily used and has more than 17 000 users, who have run more than 1 50 000 jobs in the last few years.

FFT based approaches have been successfully applied to rigid protein systems, however, they are not directly applicable to flexible systems, such as peptide-protein interactions. These transient protein complexes are important as they make up nearly half of protein interactions in the cell. To account for increased flexibility of peptides, existing global peptide docking methods instead use Monte Carlo and Molecular Dynamics-based approaches (Ben-Shimon and Niv, 2015; Dagliyan *et al.*, 2011; Kurcinski *et al.*, 2015; Raveh *et al.*, 2011; Schindler *et al.*, 2015; Trellet *et al.*, 2013; Yan *et al.*, 2016). While useful, most of these approaches require inclusion of computationally expensive refinement to obtain CAPRI quality models, and thus are too resource demanding for efficient server implementation. Template based approaches on the other hand are much faster (Lee *et al.*, 2015), but are limited to cases with available templates.

It turns out that peptides in transient complexes have particular properties that nevertheless renders the peptide-protein problem amenable to an FFT based approach. In the majority of peptide–protein interactions, a protein domain interacts with a short linear peptide motif. For example, cyclins recognize the RXL motif, where R (arginine) and L (leucine) are fixed as amino acids, and X represents any amino acid. It has been shown experimentally that motif regions are limited to a small ensemble of potential conformations within a protein environment (Wang *et al.*, 2013). This observation enables the use of FFT for our docking method, as we can dock each conformation in this small ensemble.

The key idea of our approach is to generate this ensemble by searching for proteins containing the motif of interest in the Protein Data Bank (PDB) (Berman *et al.*, 2000) and extract the regions that match the motif. Conceptually similar approaches of mining fragments from the PDB have been previously used for protein structure prediction (Simons *et al.*, 1997). While these approaches were based on sequence and secondary structure similarity, we use *motif* based fragment extraction to focus on a relevant subset of conformations, taking advantage of the specific features of the motif docking challenge. By combining this resulting fragment library with systematic Fast Fourier Transform (FFT) grid based sampling using accurate molecular mechanics potentials (Kozakov *et al.*, 2006, 2014), we efficiently sample and discriminate near native docked peptide models with success rates similar to domain–domain protein docking, despite significant peptide flexibility. We demonstrate this on a diverse set of domain–motif interactions, and make the method freely available as part of the protein–protein docking server ClusPro.

## 2 Materials and methods

(1) *Preparing the input structures*: The structure of the free receptor is represented as an independent binding unit that is defined as either a single domain, or repeated, non-decomposable domains (Lavi *et al.*, 2013). Unstructured terminal tails are removed. On the peptide side we start with a peptide sequence that covers the initial motif, and expand the sequence if the original motif is too short (less than 5 residues). We further generalize it by introducing wildcards based on motif information, or restrict it by further expansion, as detailed in SI methods. The generalization protocol is iterative, based on available PDB information, to ensure reasonable structural coverage (i.e. a library of 100–1000 conformations).

(2) *Clustering of fragments*: In the next step the fragments are clustered using a greedy algorithm and a stringent clustering radius of 0.5 Å. The cluster center of each of the top 25 clusters is retained for docking in the next step [Similar to the 25 top-scoring fragments used in Rosetta *ab initio* modeling (Simons *et al.*, 1997)].

(3) *Docking of peptide fragments*: Each of the (up to 25) fragments is docked to the receptor structure using an FFT based sampling protocol (see SI). The top 250 results of each run are combined into one ensemble.

(4) *Selection of models*: The ensemble of models is clustered using a cluster radius of 3.5 Å, which was chosen to represent the resolution of basins of attraction, and the resulting clusters are ranked according to cluster size (see SI). Representatives of the top-ranking selected clusters are further locally minimized using CHARMM (Brooks *et al.*, 2009), and provided as predictions. Solutions that overlap with domain-domain interfaces are removed.

Overview of the protocol is presented in Supplementary Figure S1.

## 3 Docking performance

To demonstrate the efficiency and accuracy of this approach, we model diverse protein domain-motif interactions from the PeptiDB v2 dataset for which the interacting motif has been reported (Lavi *et al.*, 2013; London *et al.*, 2010). Additionally, we validate the protocol on an independent set of domain-motif complexes recently published in the PDB distinct from PeptiDB v2. We note that to prevent inherent bias in modeling, structures of proteins with more than 30% sequence identity to the target are excluded from the search for motif backbones, and only information about sequence motifs available before publication of the solved complex structure is included.

Using a CAPRI-inspired threshold for success, namely defining a near-native conformation if the peptide lies within 4.0 Å backbone RMSD of the native peptide bound to the receptor (i.e. the CAPRI criterion for an acceptable peptide-protein docking prediction), a near-native peptide conformation is found among the top 10 PeptiDock predictions for 11 of the 16 complexes, and all apart from two cases are identified among the top 20 clusters. Similar performance is obtained for the additional validation set: for 4 out of 5 complexes, a conformation similar to bound is extracted using the motif based search, and for 3 out of the 5 cases a near-native structure is ranked first. The overall detailed assessment of PeptiDock performance is provided in Supplementary Table S1, and comparison of docked poses to crystal conformations are shown in Supplementary Figure S2. Comparison with another global peptide docking protocol, CABS-dock (Kurcinski *et al.*, 2015) which uses *ab-initio* folded peptide conformations rather than PDB fragments, indicates that fragment based backbone sampling provides docking models of higher accuracy (Supplementary Table S2).

**Fig. 1:**
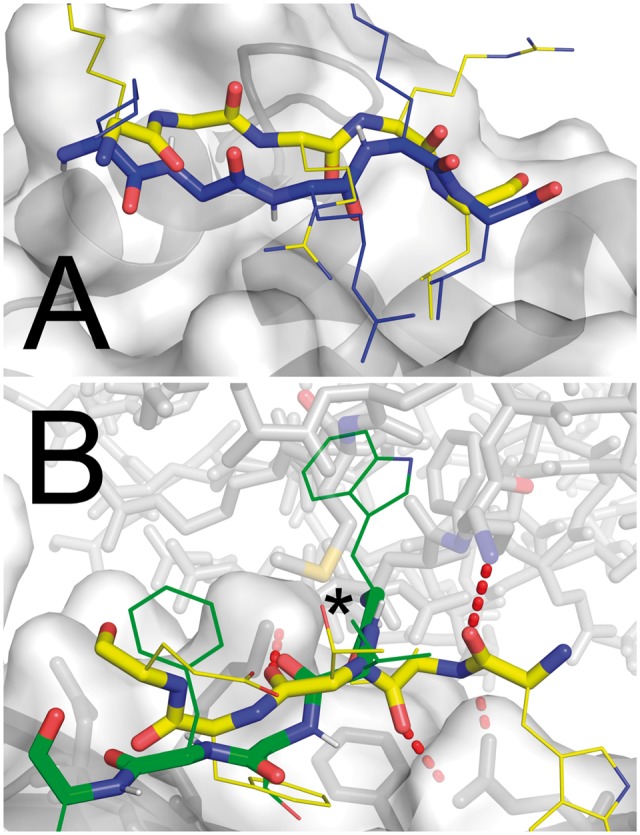
Examples for models generated by PeptiDock rigid body docking of peptides to a receptor. Receptor structures are shown in light grey. Silver structures represent the native peptide pose. (A) A peptide derived from CDC6 with the sequence motif KGRRL is successfully docked to cyclin. The third ranked prediction (dark grey) produces an acceptable accuracy result (1.9Å backbone RMSD; apo/holo PDB IDs: 1H1R/2CCH). (B) No near native structure is sampled using the standard energy function weight set when a peptide derived from synaptojanin is docked to the ap2 adaptor (apo/holo PDB IDs: 1B9K/2VJ0). Nevertheless, a 4.0Å RMSD model (gray) is produced when an electrostatics-favored coefficient set is used in place of the standard weight set. This can be explained by the fact that this interaction is dominated by several hydrogen bonds of the peptide backbone (dotted line) in the native complex, but lacks strong hydrophobic interactions with the aromatic side chains, as well as by crystal contacts in the bound conformation (shown in opaque gray sticks) that stabilize the solved peptide conformation. The hydrophobic V6 points into the “solvent”, but actually contacts the symmetry mate (interaction is marked with *; see Figure S3 for more details) (Color version of this figure is available at *Bioinformatics* online.)

Figure 1 shows an example of a successful case and a challenging case. For the latter, the native complex forms hydrogen bonds between the peptide backbone and protein side chains, but lacks strong hydrophobic interactions with the aromatic side chains. The hydrophobic valine points into the solvent (forming crystal contacts with a symmetry mate in the solved structure). Interestingly, in this and in the one additional case for which no near-native structure was sampled (2YNNA), considerable improvement was obtained by using an electrostatic-driven potential (Supplementary Table S3), indicating that scoring rather than sampling limits performance in these interactions that are dominated by electrostatic attraction (and crystal contacts).

## 4 Conclusions

We demonstrate remarkable performance in deterministic global *ab-initio* docking of peptides to protein domains using an approach that capitalizes on the relationship between sequence and structural motifs and employs accurate FFT based sampling. Results are shown for a set of domain-motif interactions. It is important to note that this set was derived from the generic diverse peptide-protein benchmark set PeptiDB v2 (Lavi *et al.*, 2013), which was designed with no motif information in mind. Nevertheless, we were able to find motif information in the literature for 16 of 21 cases in the PeptiDB v2 set, which demonstrates that motif information is generally available for a broad class of protein peptide interactions. The resulting protocol takes roughly 500 CPU hours per simulation, which translates to less than 2 hours on a cluster of 20 16-core machines. The method is freely available as part of the ClusPro protein-docking server.

## Funding

This work was supported by National Science Foundation (NSF) Grants AF 1527292 and DBI 1458509, US Israel Binational Science Foundation Grant 2009418,2015207, and European Research Council (ERCstG 310873).

*Conflict of Interest*: none declared.

## Supplementary Material

Supplementary Data
