# Supplementary material for: ClusPro PeptiDock: efficient global docking of peptide recognition motifs using FFT
Source: Bioinformatics. 2017 Apr 18;33(20):3299–301. doi: 10.1093/bioinformatics/btx216 (PMC5860028; doi:10.1093/bioinformatics/btx216)
Supplement: Supplementary Data [file btx216_supplement_final.pdf]

Supporting material for:

ClusPro PeptiDock: Efficient global docking of peptide recognition motifs using FFT.

Kathryn A Porter[1], Bing Xia[1], Dmitri Beglov[1], Tanggis Bohnuud[1], Nawsad Alam[2], Ora Schueler-Furman[2]*, Dima Kozakov[3,4]*
[1]Department of Biomedical Engineering, Boston University, Boston MA 02215 and [2]Department of Microbiology, Hebrew University, Jerusalem Israel, and [3]Department of Applied Mathematics and Statistics, Stony Brook University, Stony Brook NY 11794 and [4]Laufer Center for Physical and Quantitative Biology, Stony Brook University, Stony Brook NY 11794

SUPPLEMENTARY MATERIALS CONTENT

Supplementary Figure S1-S3
Supplementary Tables S1-S6
Supplementary Methods

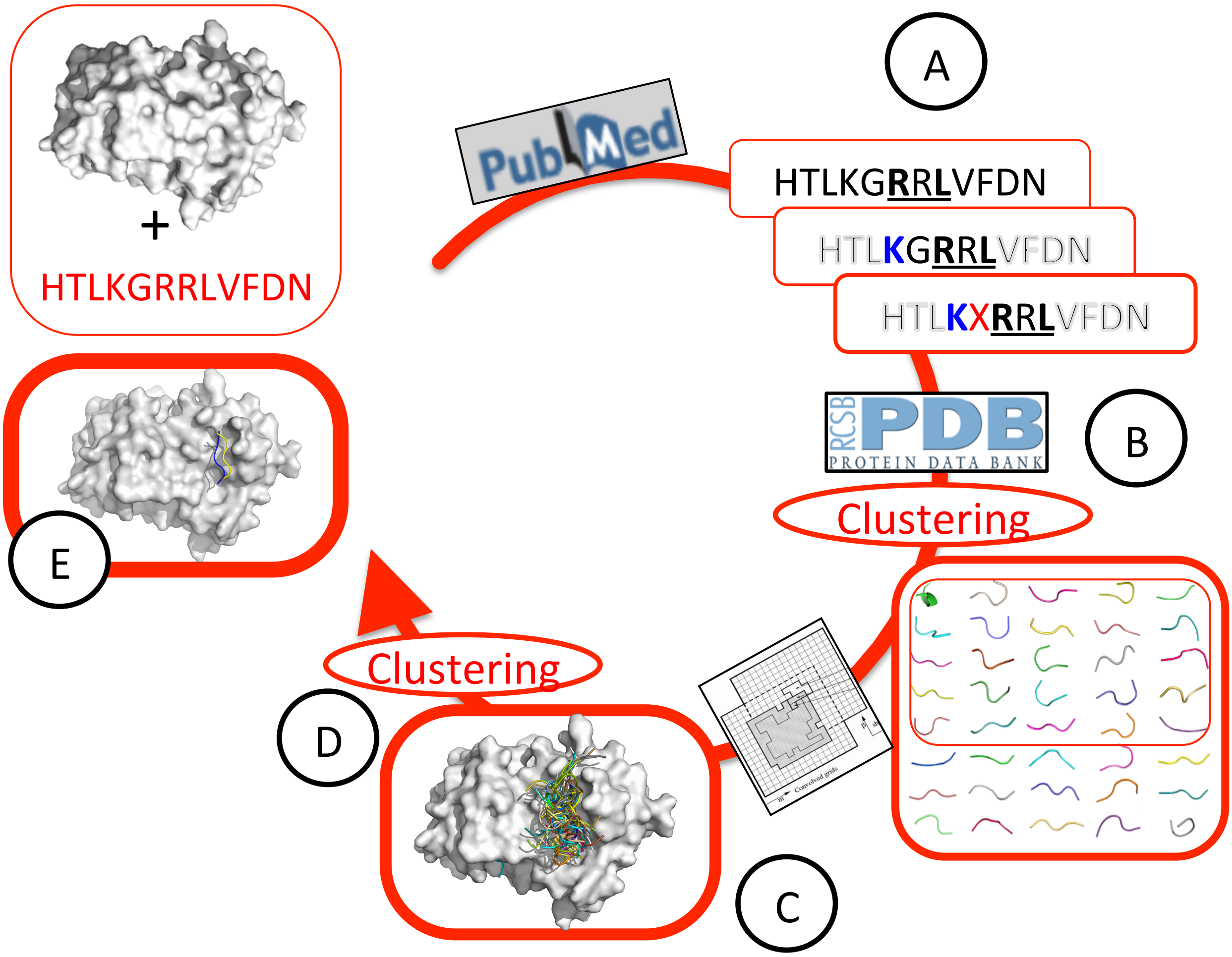

Figure S1 . Overview of the domain-motif docking protocol
Flowchart of protocol. Starting from a given (free) receptor structure and a peptide sequence (upper left), the binding motif is extracted in a first step, using information from literature and/or the ELM database . (A) This initial motif is then extended using the rules described in SI: For the specific example shown here, namely the docking of a cdc6-derived peptide HTLKGRRLVFDN to cyclin A, the motif reported in ELM is R.L (Arginine, followed by any amino acid, and a Leucine). We extended this motif to a pentamer, by extending in the n-terminal direction towards a polar residue, KGRRL. Since this motif is found only 9 times in the PDB, we made it more general by introducing a wild-card, at the smallest residue, G, resulting in KXRRL. This motif was found frequently enough to proceed. (B) We extracted the matching fragments from the PDB and clustered them with a 0.5Å RMSD cluster threshold. (C) Representatives from the top-25 Largest clusters were then each docked to the receptor structure. (D) Pooling all solutions and Clustering with a 3.5Å RMSD threshold resulted in a set of predictions that were further minimized (SI). (E) The 3rd ranked cluster (according to cluster size) contains a near-native solution of 1.9Å RMSD (native / predicted structures shown in magenta / yellow cartoons, respectively).

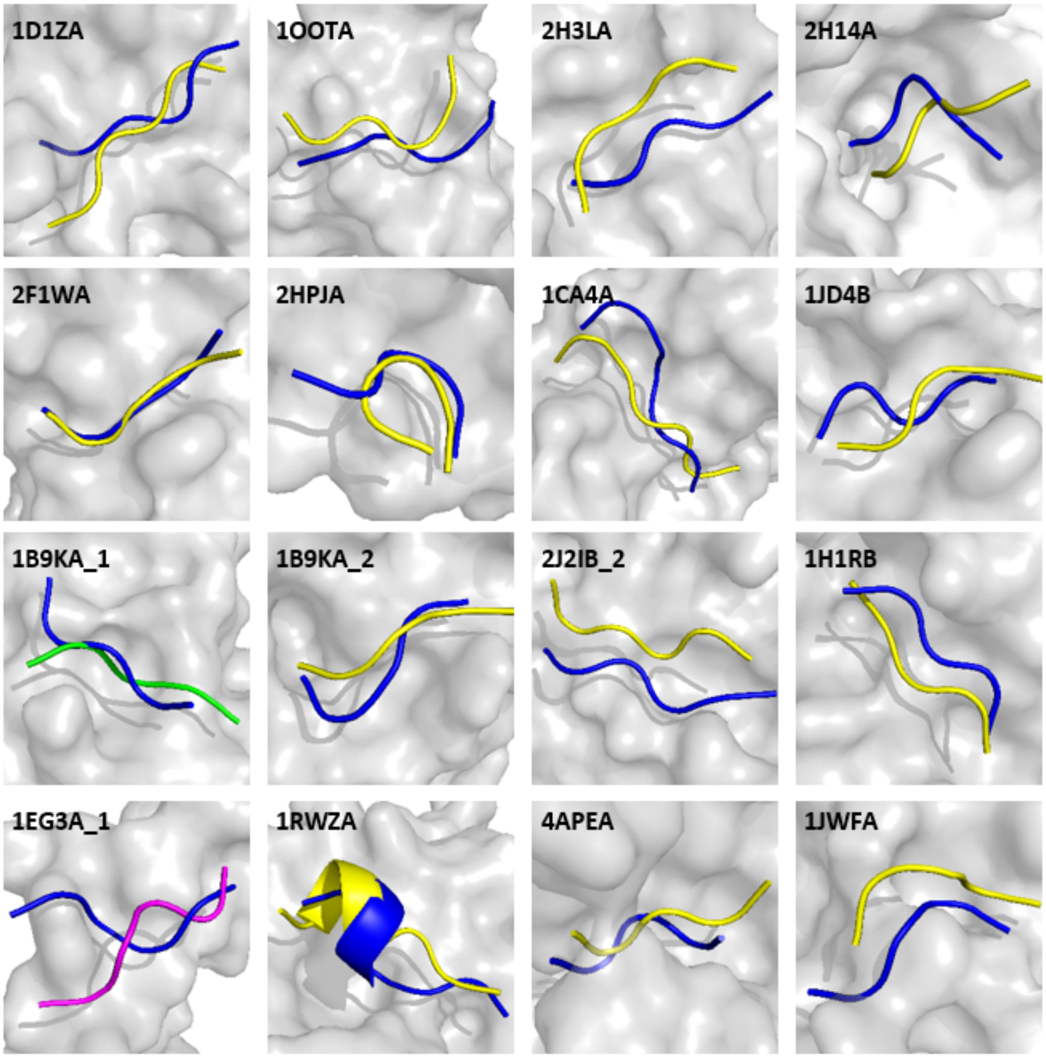

Figure S2. Modeled protein-peptide complexes. Blue is the crystal, native pose, yellow is the acceptable accuracy model. Pink shows the closest non-acceptable accuracy model produced by the approach. Green depicts the acceptable accuracy model for a case (PDB ID 2VJ0) in which only the electrostatics coefficient set gave a strong result.

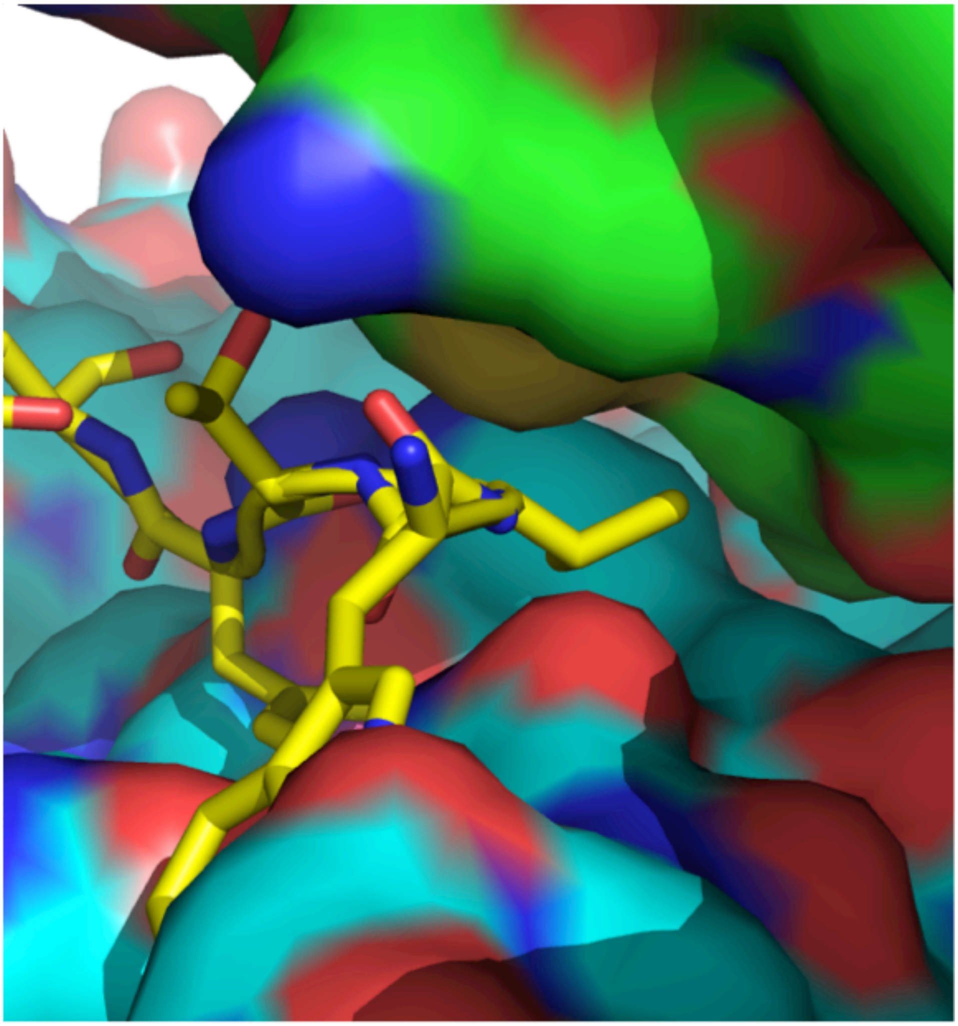

Figure S3. Crystall symmetry interface of the alpha-adaptin WVTFE interaction. Two crystal symmetry mates of the bound protein peptide complex (PDB 2VJ0) are shown in blue and green, and the peptide chain is colored in yellow. The view is focused on tight packing of Val 6 of the peptide with methionine 914 of the crystal symmetry partner, which further suggests that the bound peptide pose is most probably strongly affected by the crystal conditions, and therefore not expected to be reproduced by predictions that do not take crystal symmetry into account.

SUPPLEMENTARY METHODS

Supplementary Overview of the algorithm. Figure S1 demonstrates the steps of our protocol on an example application, the interaction between cyclin (structure of free cyclin, PDB ID 1H1R (Davies, et al., 2002)) and a peptide derived from CDC6 (HTLKGRRLVFDN) (Cheng, et al., 2006) (structure of the complex, PDB ID 2CCH (Cheng, et al., 2006)). The RxL peptide motif (Arginine, followed by any amino acid, and Leucine) was defined based on a literature search (Cheng, et al., 2006). We use a set of rules (defined below in the 'Buildup of motif' section) to extend and refine this initial motif, until a search in the PDB results in a comprehensive set of fragment hits (note that for protocol validation homologs of complex structures are, of course, excluded). In this example (see **Figure S1A)**, the peptide sequence covering the initial motif (i.e. RRL) was first extended in the N-terminal direction towards a polar residue, to yield a pentamer sequence KGRRL (applying rule **Sp**). Since this motif is found only nine times in the PDB, we made it more general by introducing a wild-card at the smallest residue, G, to obtain KXRRL (rule **E**). This motif is found frequently enough to proceed to the next step (in 472 PDB structures, homologs of the solved CDC6-cyclin structure (PDB ID 2CCH) excluded). Once the docking motif is defined, we extract the matching fragments from the PDB (1051 fragments) and cluster them with a 0.5Å RMSD cluster threshold (resulting in 40 clusters for this example, see **Figure S1B** and **Table S6A**). Representatives from the top 25 largest clusters are then each docked to the receptor structure (**Figure S1C**). All solutions are pooled and clustered with a 3.5Å RMSD threshold, and representatives of each cluster are further minimized to produce the final 100 models (**Figure S1D** and **Table S6B**). In the cyclin-CDC6 peptide example, the third ranked model lies within 1.9Å RMSD (**Figure 1E)**. This can be seen more clearly in **Figure S1**.

Supplementary details on the components of the protocol.

Buildup of motif: Successful definition of a good motif for peptide fragment selection is the critical step of our protocol: A general, non-biased protocol should define a motif that is both loose enough to provide good coverage, and informative enough to enrich for relevant conformations. We start from the peptide sequence of interest and a known motif, and apply the following rules (**Figure S1A**; see **Table S5** for application of these rules in the predictions reported here): (1) Start (**S**): Start with a peptide sequence of minimal length of 5 residues (to allow for a motif of 4 and more residues and one or more wild cards if necessary). This sequence should cover the initial motif, and if needed be extended by including additional positions in the peptide sequence. The preferential direction of extension is defined based on the type of residues, according to the following priority: (**Sp**) Polar, (**Sa**) Aromatic, and (**So**) other residues. Small amino acids (GSTA) are not considered for extension, except as a bridge to the next extended residue (e.g. extension of PXQ motif to PQQATD for the peptide PQQATDD, leading to a 6 residue long starting motif), or if this is the only possible option to extend the motif to the minimal length. This initial sequence will usually result in very few fragment hits in the PDB, and we therefore expand the motif in the following step(s). (2) Expand (**E**): Insert wildcards back (X, or redundant positions of the motif), starting with the smallest residues. Refrain from introducing adjacent wild cards if possible, and do not introduce X at the termini of the peptide. (3) Large (**L**): If more than 1000 hits to PDB structures are found, introduce specific residues back into the motif, starting with the largest residues. If this does not help, try to extend, if possible. (4) Stop when there are between 100 and 1000 hits to PDB structures (or more if further extension of motif is not possible). (5) Complement F/Y (**F**): F & Y show very similar conformational preferences in the backbone dependent rotamer libraries (Ting, et al., 2010). Once the motif has been designed and the set of fragments has been extracted, the amino acid sequence is changed back to the actual peptide sequence (using a backbone-dependent rotamer library(Dunbrack and Karplus, 1993)).

Docking: Global sampling by fast Fourier transform (FFT) correlation
Docking was performed as detailed before (Kozakov, et al., 2013). In short, to fully explore the conformational space of rigid body orientations between a given peptide conformer and the receptor, we perform exhaustive evaluation of an energy function in the discretized space of mutual orientations

of the protein and peptide using fast Fourier transform (FFT) correlation approach (Kozakov, et al., 2006). The center of mass of the receptor protein is fixed at the origin of the coordinate system, whereas the peptide conformer, defined as the ligand, is rotated and translated. The translational space is represented as a grid of 1.0 Å displacements of the ligand center of mass, and the rotational space is sampled using 70,000 rotations based on a deterministic layered Sukharev grid sequence, which quasi-uniformly covers the space. The energy expression used for the FFT based sampling includes a simplified van der Waals energy E_{vdw} with attractive (E_{attr}) and repulsive (E_{rep}) contributions, the electrostatic interaction energy E_{elec}, and a statistical pairwise potential E_{pair}, representing other solvation effects (Chuang, et al., 2008):

$$E = E_{vdw} + w_2 E_{elec} + w_3 E_{pair}$$

The individual energy terms are calculated as $E_{vdw} = E_{attr} + w_1 E_{rep}$, $E_{elec} = \Sigma_i \Sigma_j [q_i q_j / \{r^2 + D^2 \exp(-r^2/4D^2)\}^{1/2}]$, and $E_{pair} = \Sigma_i \Sigma_j \varepsilon_{ij}$, where r is the distance between atoms i and j, D is an atom-type independent approximation of the generalized Born radius, and ε_{ij} is a pairwise interaction potential between atoms i and j. Two options of weights sets are used: The original set (w_1=1.3, w_2=160 and w_3=2.6) and a set of weights that was recently shown to improve performance for polar-dominated interactions (w_1=4, w_2=600, w_3=0: the pairwise potential is omitted, and consequently the relative electrostatic contribution is increased (Kozakov, et al., 2013)). For consistency, we use here the original set of weights, and report on improvement for polar interactions in the text and **Table S3**. All energy expressions are defined on the grid. In order to evaluate the energy function E by FFT, it must be written as a sum of correlation functions. The first two terms, E_{vdw} and E_{elec}, satisfy this condition, whereas E_{pair} is written as a sum of a few correlation functions, using an eigenvalue-eigenvector decomposition (Kozakov, et al., 2006). For each rotation, this expression can be efficiently calculated using P forward and one inverse Fast Fourier transforms. The calculations are performed for each of the 70,000 rotations, and one lowest-energy translation for each rotation is retained.

Clustering algorithm

For k structures, we calculate the k × k matrix of pairwise backbone RMSDs. We count the number of neighbors each structure has within a defined cluster radius. The members of the largest cluster are removed from the pool of structures, and the procedure is repeated until no structures remain, resulting in clusters ranked according to their size (Kozakov, et al., 2005). The cluster radius cutoff is set to 0.5Å for the clustering of backbone fragments (a 2.0Å cutoff was found to result in a too small number of clusters), and to 3.5Å for the clustering of docking solutions. The latter represents the assumed radius of attraction for peptide-protein docking.

Scoring according to cluster size

In biophysical terms, clusters represent isolated, highly populated low energy basins of the energy landscape, and the large clusters are thus more likely to include native structures [8]. The globally sampled conformational space can be considered as a canonical ensemble with the partition function $Z = \Sigma_j \exp(-E_j/RT)$, where we sum the associated energy E_j over all poses j. For the kth cluster, the partition function is given by $Z_k = \Sigma_j \exp(E_j/RT)$, where the sum is restricted to poses within the cluster. Based on these values, the probability of the kth cluster is given by $P_k = Z_k/Z$. Since the low energy structures are selected from a relatively narrow energy range, and the energy values are calculated with considerable error, it is reasonable to assume that these energies do not differ, *i.e.,* E_j=E for all j in the low energy clusters. This simplification implies that $P_k = \exp(-E/RT) \times N_k/Z$, and thus the probability P_k is proportional to N_k, where N_k is the number of structures in the kth cluster.

Minimization of final structures

For minimization we use the polar hydrogen PARAM19 like forcefield with CHARMM (Brooks, et al., 2009). The protocol consists of 500 steps of unconstrained Adapted Basic Newton-Raphson (ABNR) minimization, where both protein and peptide are free to move, followed by the restoration of crystal protein coordinates, and 1000 steps of ABNR minimization of the peptide with the fixed protein.

Table S1. Overall assessment of the motif-domain docking performance. Global docking of motifs identifies for most cases near-native peptide conformations (within 4.0Å peptide backbone RMSD) among the top-ranking predictions.

Bound [a]	Free [b]	Peptide [c]	Motif reported [d]	Motif scanned in PDB [e]	Rank [f]	RMSD [g] (Å)
**PeptiDB v2 set**						
1D4TA	1D1ZA	KSL**TIY**AQ**V**QK	TIYXX[VI] [23]	**TI**[**Y**F]XX[**VI**]	**5**	**3.7**
1SSHA	1OOTA	GPP**P**AM**P**A**R**PT	PXXPX[R/K] [24]	**P**XM**P**X**R**	**8**	**3.4**
1MFGA	2H3LA	EYLGLD**V**P**V**	VXV' [25]	LD**V**X**V**	**3**	**3.9**
2H9MA	2H14A	**AR**TKQT	γδRγ [26]	**AR**[TS]KQ	12	**3.8**
2FOJA	2F1WA	GAR**A**HS**S**	[PA]XXS [27]	R[**PA**]HX**S**	18	**1.7**
2HPLA	2HPJA	DDL**Y**G	ϕYX' [28]	DXL[**Y**F]G	**1**	**3.5**
1CZYA	1CA4A	ace-**P**Q**Q**A**T**DD	PxQ [f 29]	**P**X**Q**XXDD	**4**	**3.3**
1JD5A	1JD4B	**AIAY**FIPD	A[VTI][AP][FY] [30]	**A**[**VTI**][**AP**][**YF**][YF]	**2**	**3.5**
2VJ0A	1B9KA_1	PKG**W**VT**F**E	WXX[F/W] [32]	**W**XX[**F**Y]E	-	>6.0
2VJ0A	1B9KA_2	**FED**N**F**VP	DXF [31]	[FY]X**D**N[**F**Y]	**5**	**2.4**
2C3IB	2J2IB_2	K**R**R**RH**PSG	RXRHXS [33]	**R**X**RH**X**S**	**8**	**4.0**
2CCHB	1H1RB	HTLKG**RRL**VFDN	RXL [10]	KX**RRL**	**3**	**1.9**
1EG4A	1EG3A_1	NMTPYRS**PPPY**VP	PPXY [22]	RX**PP**X[**Y**F]	**10**	4.1
1RXZA	1RWZA	KST**Q**AT**L**ER**WF**	QXXϕXXρρ [34]	**Q**XX[**LVI**]XX**W**[**FY**]	**3**	**3.5**
1ER8E	4APEA	PFH**LL**VY	ϕϕ (E.C.3.4.23.22 [h])	H [**LVI**][**LVI**][**LVI**][YF]	**10**	**2.9**
1JWGAC	1JWFA	**D**ED**LL**HI	DXXLL' [35]	**D**XD**LL**	22	**4.0**
**"Recent PDB" set**						
4FCMB	4FCJB	SG**F**S**F**	FXFG [36]	SX[**F**Y]S[**F**Y]	36	**4.0**
3ZGCA	3ZGDA	G**D**E**ETGE**	DXETGE [37]	**D**X**ETGE**	**10**	**3.9**
4GK5E	4GK0E	S**FF**DKKRS	FF [38]	[**F**Y][**F**Y]DXK	**2**	**1.7**
4R5IA	4R5JA	NR**LLL**T	LLL [39]	NR**LLL**	**2**	**3.8**
2YNNA	2YNOB	CTF**K**T**K**TN	KXKXX' [40]	**K**T**K**XN	-	>6.0

[a] PDB id of receptor-peptide complex structure; [b] PDB id of free receptor structure, including chain, and number of domain in multi-domain proteins (according to CATH); [c] Region underlined is part of the motif; defined amino acids in the motif are in bold; [d] Motif definitions: ' - c-terminal; δ- small (A,G); γ- no bulky side chains; ϕ – hydrophobic side chain; ρ- aromatic side chain; [e] See text for definition of motif in this study; [f] Best rank of model within 4.0Å RMSD; ranks 1-10 in bold; [g] Peptide backbone RMSD; successful predictions (<= 4.0Å RMSD) are in bold; [h] Flanking cleavage site - Enzyme Nomenclature EC number(http://www.chem.qmul.ac.uk/iubmb/enzyme/)

Table S2. Comparison between performance of PeptiDock and CABS-dock.

		PeptiDock		CABS-dock	
**Case**	Docking sequence	Rank	RMSD (Å)	Rank	RMSD (Å)
**PeptiDB v2 set**					
1D4TA	TIYAQV	**5**[a]	**3.7**	2	7.9
1SSHA	PAMPAR	**8**	**3.4**	2	6.8
1MFGA	LDVPV	**3**	**3.9**	4	9.1
2H9MA	ARTKQ	4	4.9	4	4.6
2FOJA	RAHSS	5	6.6	5	13.5
2HPLA	DDLYG	**5**	**3.4**	4	4.8
1CZYA	PQQATDD	**4**	**3.3**	**1**	**3.1**
1JD5A	AIAYF	**2**	**3.5**	10	9.9
2VJ0A	WVTFE	7	27.6	7	5.3
2VJ0A	FEDNF	**5**	**2.4**	**5**	**3.3**
2C3IA	RRRHPS	**8**	**4.0**	5	5.3
2CCHB	KGRRL	**3**	**1.9**	8	7.8
1EG4A	RSPPY	10	4.1	7	4.7
1RXZA	QATLERWF	**3**	**3.5**	3	9.8
1ER8E	HLLVY	**10**	**2.9**	4	7.4
1JWGAC	DEDLL	7	4.6	6	6.6
**"Recent" PDB set**					
4FCMA	SGFSF	1	6.8	3	6.7
3ZGCA	DEETGE	**10**	**3.9**	8	5.7
4GK5E	FFDKK	**2**	**1.7**	2	4.2
4R5IA	NRLLL	**8**	**1.5**	6	11.2
2YNNA	KTKTN	6	10.2	4	4.7

[a] Predictions within 4.0Å RMSD are highlighted in bold. Note that the same input was provided, and that we use the unbound receptor structure (even though the bound pdb id is listed here; see Table S1)

Table S3. Use of electrostatic-driven potential improves performance for specific cases. Since no near-native structures were sampled for two cases (PeptiDB v2: 2VJ0 & Recent PDB: 2YNN) using the 'Normal' energy function weight set, the cases were re-docked using an electrostatic driven potential.

Bound	Free	Peptide	Motif reported	Motif used for scanning PDB	Energy function weight	Rank	RMSD (Å)
2VJ0A	1B9KA_1	PKG**W**VT**F**E	WXX[F/W] (Olesen, et al., 2008)	**W**XX[**F**Y]E	Normal	-	>6.0
					Electrostatic	**3**	**3.9**
2YNNA	2YNOB	CTF**K**T**K**TN	KXKXX' (Jackson, et al., 1990)	**K**T**K**XN	Normal	-	>6.0
					Electrostatic	**2**	**3.9**

[a] PDB id of receptor-peptide complex structure; [b] PDB id of free receptor structure, including chain, and number of domain in multi-domain proteins (according to CATH); [c] Region underlined is part of the motif; defined amino acids in the motif are in bold; [d] Motif definitions: ' - c-terminal; δ- small (A,G); γ- no bulky side chains; ϕ – hydrophobic side chain; ρ- aromatic side chain; [e] See text for definition of motif in this study; [f] Best rank of model within 4.0Å RMSD; ranks 1-10 in bold; [g] Peptide backbone RMSD; successful predictions (<= 4.0Å RMSD) are in bold; [h] Flanking cleavage site - Enzyme Nomenclature EC number(http://www.chem.qmul.ac.uk/iubmb/enzyme/)

Table S4. Set of peptide-protein complexes used in this study. We model a diverse set of 16 domain-motif interactions from the PeptiDB v2 set. The docking protocol was validated on a set of 5 motif-domain complexes recently published in the PDB. For each complex, a bound and free receptor structure is available in the PDB, and an interaction motif has been reported.

	Bound [a]	Free [b]	Peptide [c]	Motif reported [d]
**PeptiDB v2 set**				
sh2a1 (SH2)	1D4TA	1D1ZA	KSL**TIY**AQ**V**QK	TIYXX[VI] (Poy, et al., 1999)
lsb3 sla1 (SH3)	1SSHA	1OOTA	GPP**P**AM**P**A**R**PT	PXXPX[R/K] (Hou, et al., 2012)
erbB2 (PDZ)	1MFGA	2H3LA	EYLGLD**VPV**	VXV’ (Jaulin-Bastard, et al., 2001)
wdr5 (WD40)	2H9MA	2H14A	A**R**TKQT	gδRg (Schuetz, et al., 2006)
usp7 (MATH)	2FOJA	2F1WA	GAR**A**HS**S**	[PA]XXS (Sheng, et al., 2006)
p97 N-glycanase (PUB)	2HPLA	2HPJA	DDL**YG**	ϕYX’ (Smith, et al., 2007)
traf2 (TRAF)	1CZYA	1CA4A	ace-**P**Q**Q**A**T**DD	PxQ (Devergne, et al., 1996)
i-ap1 (BIR)	1JD5A	1JD4B	**AIAY**FIPD	A[VTI][AP][FY] (Srinivasula, et al., 2001)
ap2 (appendage domains)	2VJ0A	1B9KA_2	**F**E**D**N**F**VP	DXF (Brett, et al., 2002)
ap2	2VJ0A	1B9KA_1	PKG**W**VT**F**E	WXX[F/W] (Olesen, et al., 2008)
pim1 kinase (transferase domain)	2C3IA	2J2IB_2	K**R**R**RH**P**S**G	RXRHXS (Bullock, et al., 2005)
cdk2 cyclin	2CCHB	1H1RB	HTLKG**R**R**L**VFDN	RXL(Cheng, et al., 2006)
dystrophin (WW)	1EG4A	1EG3A_1	NMTPYRS**PP**P**Y**VP	PPXY (Chen and Sudol, 1995)
pcna	1RXZA	1RWZA	KST**Q**AT**L**ER**WF**	QXXϕXXρρ (Warbrick, 1998)
endothiapepsin	1ER8E	4APEA	PFH**LLV**Y	ϕϕ (E.C.3.4.23.22 [e])
gga1 (VHS)	1JWGAC	1JWFA	**D**ED**LL**HI	DXXLL’ (Chen, et al., 1997)
**“Recent PDB” set**				
G3BP1 (NF2-like domain)	4FCMA	4FCJB	SG**F**S**F**	FXFG (Clarkson, et al., 1996)
KEAP1 (Kelch)	3ZGCA	3ZGDA	G**D**E**ETGE**	DXETGE (Kobayashi, et al., 2002)
Rev1 (C-terminal domain)	4GK5E	4GK0E	S**FF**DKKRS	FF (Ohashi, et al., 2009)
DNAK (C-terminal domain)	4R5IA	4R5JA	NR**LLL**T	LLL (Rudiger, et al., 1997)
COPI (WD40) [f]	2YNNA	2YNOB	CTF**K**T**K**TN	KXKXX' (Jackson, et al., 1990)

[a] PDB id of receptor-peptide complex structure; [b] PDB id of free receptor structure, including chain, and number of domain in multi-domain proteins (according to CATH); [c] Region underlined is part of the motif; defined amino acids in the motif are in bold; [d] Motif definitions: ‘ - c-terminal; δ- small (A,G); γ- no bulky side chains; ϕ – hydrophobic side chain; ρ- aromatic side chain; [e] Flanking cleavage site: Enzyme Nomenclature EC number (http://www.chem.qmul.ac.uk/iubmb/enzyme/); [f] Same peptide binding domain (WD40) as in PeptiDB v2 set, but different peptide motif.

Table S5. Definition of sequence motifs for the extraction of fragments from the PDB. For each peptide sequence, docking motif selection is shown in a step by step fashion, following the motif building rules detailed in the above ‘Buildup of motif’ section.

Peptide	Motif reported [a]	b	c										
**PeptiDB v2 set**													
KSLTIYAQV QK	TIYXX[VI]	**S** ➔	TIYAQV (2)	**E** ➔	TIYXQV (2)	**E** ➔	TIYXQ[VI] (24)	**E** ➔	TIYXX[VI] (208)	**F** ➔	**TI[YF]XX[VI] (686)**		
GPPPAMPA RPT	PXXPX[R /K]	**S** ➔	PAMPAR (0)	**E** ➔	PAMPXR (0)	**E** ➔	**PXMPXR (107)**						
EYLGLDVP V	VXV’	**Sp** ➔	LDVPV (33)	**E** ➔	**LDVXV (773)**								
ARTKQT	γδRγ	**Sp** ➔	ARTKQ (234)										
GARAHSS	[PA]XXS	**Sp** ➔	RAHSS (40)	**E** ➔	R[PA]HSS (43)	**E** ➔	**R[PA]HXS (222)**						
DDLYG	φYX’	**Sp** ➔	DDLYG (34)	**E** ➔	DXLYG (457)	**F** ➔	**DXL[YF]G (1041)**						
ace-PQQATDD	PXQ	**Sp** ➔	PQQATD (0)	**E** ➔	PQQXTD (0)	**E** ➔	PXQXTD (81)	**E** ➔	PXQXXD (2801)	**L** ➔	**PXQXXDD (222)**		
AIAYFIPD	A[VTI][AP][FY]	**Sa** ➔	AIAYF (8)	**E** ➔	AI[AP]YF (17)	**E** ➔	A[VTI][AP]YF (39)	**E** ➔	A[VTI][AP][FY]F (144)	**F** ➔	**A[VTI][AP][FY][FY] (322)**		
FEDNFVP	DXF	**Sa** ➔	FEDNF (3)	**E** ➔	FXDNF (142)	**F** ➔	**[FY]XDN[FY] (550)**						
PKGWVTFE	WXX(F/W)	**Sp** ➔	WVTFE (0)	**E** ➔	WVXFE (28)	**E** ➔	WXXFE (745)	**F** ➔	**WXX[FY]E (1733)**				
KRRRHPSG	RXRHXS	**S** ➔	RRRHPS (6)	**E** ➔	RRRHXS (6)	**E** ➔	**RXRHXS (198)**						
HTLKGRRL VFDN	RXL	**Sp** ➔	KGRRL (8)	**E** ➔	**KXRRL (475)**								
NMTPYRSP PPYVP	PPXY	**Sp** ➔	RSPPPY (0)	**E** ➔	RXPPPY (1)	**E** ➔	RXPPXY (117)	**F** ➔	**RXPPX[YF] (230)**				
KSTQATLE RWF	QXXφXX ρρ	**S** ➔	QATLERWF (2)	**E** ➔	QXTLERWF (2)	**E** ➔	QXT[LVI]ERWF (2)	**E** ➔	QXT[LVI]EXWF (4)	**E** ➔	QXX[LVI]EXW[FY] (45)	**E** ➔	**QXX[LVI]XXW[FY] (430)**
PFHLLVY	φφ flanking cleavage site	**Sa** ➔	HLLVY (2)	**E** ➔	HL[LVI]VY (5)	**E** ➔	HL[LVI][LVI]Y (32)	**E** ➔	H[LVI][LVI][LVI]Y (114)	**F** ➔	H[LVI][LVI][LVI][YF] (276)		

DEDLLHI	DXXLL'	**S** ➔	DEDLL (51)	**E** ➔	**DXDLL (832)**		

"Recent PDB" set

SGFSF	FXFG	**S** ➔	SGFSF (36)	**E** ➔	**SXFSF (293)**	**F** ➔	**SX[FY]S[FY] (1221)**
GDEETGE	DXETGE	**S** ➔	DEETGE (6)	**E** ➔	**DXETGE (173)**		
SFFDKKRS	FF	**Sp** ➔	FFDKK (9)	**E** ➔	**FFDXK (142)**	**F** ➔	**[FY][FY]DXK (597)**
NRLLLT	LLL	**Sp** ➔	**NRLLL (145)**				
CTFKTKTN	KXK	**Sp** ➔	KTKTN (12)	**E** ➔	**KTKXN (230)**		

[a] Motif definitions: ' - c-terminal; δ - small (A,G); γ - no bulky side chains; φ – hydrophobic side chain; ρ - aromatic side chain; [b] Rule applied to refine motif (See text for the definition of these rules);[c] Resulting motif (in parentheses: number of PDB structures with matching fragments – aim for [100 .. 1000])

Table S6. Example – prediction of binding of CDC6 derived peptide to cyclin. (A) Fragments extracted from PDB using the KXRRL motif, clustered according to 0.5Å RMSD cutoff, ranked according to cluster size (1051 fragments were clustered into 40 clusters; the top 25 were used for docking). The source PDB of the cluster center, as well as its RMSD to the native peptide conformation in the complex are indicated in the 3rd and 4th column, respectively. **(B)** Docking results: Models were clustered using a 3.5Å RMSD threshold into 100 clusters, and ranked according to cluster size. RMSD values to the native conformation are given both for the structure before and after local minimization in the 3rd and 4th column, respectively. The 3rd ranking cluster is 1.9Å RMSD away from native structure.

(A)

Cluster	Cluster Size	Source	RMSD – $C\alpha$ (Å)
1	417	2B8P	2.91
2	256	3P50	2.04
3	87	1G3I	0.44
4	63	3UKX	0.85
5	26	2CQS	1.98
6	19	1AGI	2.69
7	18	1JVB	1.89
8	16	1BL9	1.65
9	14	3VZB	2.17
10	9	1A25	1.38
11	8	2HPI	1.63
12	7	2Z11	1.85
13	6	1PML	2.72
14	6	1YEW	2.35
15	6	1WN1	0.44
16	6	3TFH	2.64
17	5	3A5Z	1.05
18	5	4M59	1.32
19	4	1JKG	2.03
20	4	2YN9	2.11
21	4	3HM0	0.85
22	4	4GQY	2.01
23	3	4LQS	2.05
24	2	3KH5	1.8
25	2	1YRP	1.68
26	2	2E61	0.6
27	2	2FEF	1.44
28	2	2H1E	2.53
29	2	3A32	0.72
30	2	3C1A	1.39

31	2	3IL0	1.76
32	2	3KTW	0.98
33	2	3N05	1.77
34	2	3QWU	1.53
35	2	3Q6S	1.06
36	2	3SL7	1.89
37	2	4B0R	0.91
38	2	4GQV	1.72
39	2	4M4W	2.89
40	1	1G4A	0.65

(B)

**Cluster Center**	**Cluster Size**	**RMSD (Å)**	**RMSD after Minimization (Å)**
1	714	8.8	9.0
2	559	4.6	4.7
3	424	2.7	1.9
4	274	5.0	4.8
5	181	8.0	8.8
6	171	10.0	9.5
7	169	10.0	10.5
8	169	35.7	36.1
9	145	4.4	3.3
10	145	4.9	6.3
11	122	7.7	8.4
12	117	4.3	5.6
13	112	5.4	5.0
14	110	4.5	5.3
15	105	35.0	34.5
16	104	5.0	4.4
17	101	8.0	9.3
18	101	7.8	7.8
19	94	5.3	4.8
20	86	4.6	5.0
21	83	9.7	9.8
22	83	34.1	34.5
23	75	9.8	9.8
24	72	10.8	10.0
25	68	7.0	4.5
26	68	5.1	6.1
27	61	4.2	4.2

28	60	7.4	8.4
29	59	5.2	5.4
30	58	5.1	4.6
31	54	9.3	9.5
32	49	33.9	35.3
33	48	36.3	36.3
34	47	10.2	9.8
35	46	7.7	6.7
36	43	37.6	37.8
37	43	8.4	8.3
38	42	10.2	9.3
39	37	9.2	9.4
40	35	3.6	2.8
41	35	32.1	32.7
42	35	31.2	31.8
43	32	5.8	5.9
44	32	9.8	10.7
45	30	9.1	8.7
46	29	35.9	36.3
47	29	12.5	12.3
48	28	9.2	9.2
49	28	34.4	35.8
50	27	34.7	35.4
51	26	35.4	36.0
52	25	7.5	7.8
53	25	5.5	6.4
54	25	31.8	33.9
55	24	6.3	6.6
56	23	5.4	4.1
57	23	8.2	8.4
58	22	8.5	8.8
59	22	11.7	11.3
60	21	7.3	7.3
61	21	25.4	26.2
62	21	38.8	37.1
63	20	7.9	9.0
64	20	36.0	35.4
65	20	38.8	37.2
66	19	5.5	5.7
67	19	8.0	8.6
68	18	33.5	35.4

69	18	9.1	8.7
70	17	7.8	8.0
71	17	35.7	36.6
72	16	7.4	6.2
73	15	8.9	8.7
74	14	6.9	6.3
75	14	8.0	7.9
76	13	9.1	10.5
77	13	35.7	36.6
78	13	5.7	8.0
79	12	4.7	4.3
80	10	7.9	8.1
81	10	36.3	35.3
82	9	5.2	3.8
83	9	7.8	7.4
84	9	33.2	35.2
85	9	8.5	8.2
86	9	8.0	8.0
87	8	8.9	8.4
88	7	35.9	36.3
89	7	13.0	12.8
90	6	5.9	5.6
91	5	35.1	35.7
92	4	10.0	9.5
93	4	33.3	34.5
94	4	5.1	5.8
95	4	33.0	34.1
96	4	9.9	10.6
97	4	6.2	6.3
98	3	9.4	11.0
99	3	8.9	9.6
100	1	5.2	5.9

References:

Brett, T.J., Traub, L.M. and Fremont, D.H. Accessory protein recruitment motifs in clathrin-mediated endocytosis. *Structure* 2002;10(6):797-809.
Brooks, B.R., *et al.* CHARMM: The biomolecular simulation program. *Journal of Computational Chemistry* 2009;30(10):1545-1614.
Bullock, A.N., *et al.* Structure and substrate specificity of the Pim-1 kinase. *J Biol Chem* 2005;280(50):41675-41682.
Chen, H.I. and Sudol, M. The WW domain of Yes-associated protein binds a proline-rich ligand that differs from the consensus established for Src homology 3-binding modules. *Proc Natl Acad Sci U S A* 1995;92(17):7819-7823.
Chen, H.J., Yuan, J. and Lobel, P. Systematic mutational analysis of the cation-independent mannose 6-phosphate/insulin-like growth factor II receptor cytoplasmic domain. An acidic cluster containing a key aspartate is important for function in lysosomal enzyme sorting. *J Biol Chem* 1997;272(11):7003-7012.
Cheng, K.Y., *et al.* The role of the phospho-CDK2/cyclin A recruitment site in substrate recognition. *J Biol Chem* 2006;281(32):23167-23179.
Chuang, G.Y., *et al.* DARS (Decoys As the Reference State) potentials for protein-protein docking. *Biophys J* 2008;95(9):4217-4227.
Clarkson, W.D., Kent, H.M. and Stewart, M. Separate binding sites on nuclear transport factor 2 (NTF2) for GDP-Ran and the phenylalanine-rich repeat regions of nucleoporins p62 and Nsp1p. *J Mol Biol* 1996;263(4):517-524.
Davies, T.G., *et al.* Structure-based design of a potent purine-based cyclin-dependent kinase inhibitor. *Nat Struct Biol* 2002;9(10):745-749.
Devergne, O., *et al.* Association of TRAF1, TRAF2, and TRAF3 with an Epstein-Barr virus LMP1 domain important for B-lymphocyte transformation: role in NF-kappaB activation. *Mol Cell Biol* 1996;16(12):7098-7108.
Dunbrack, R.L., Jr. and Karplus, M. Backbone-dependent rotamer library for proteins. Application to side-chain prediction. *J Mol Biol* 1993;230(2):543-574.
Hou, T., *et al.* Characterization of domain-peptide interaction interface: prediction of SH3 domain-mediated protein-protein interaction network in yeast by generic structure-based models. *J Proteome Res* 2012;11(5):2982-2995.
Jackson, M.R., Nilsson, T. and Peterson, P.A. Identification of a consensus motif for retention of transmembrane proteins in the endoplasmic reticulum. *EMBO J* 1990;9(10):3153-3162.
Jaulin-Bastard, F., *et al.* The ERBB2/HER2 receptor differentially interacts with ERBIN and PICK1 PSD-95/DLG/ZO-1 domain proteins. *J Biol Chem* 2001;276(18):15256-15263.
Kobayashi, M., *et al.* Identification of the interactive interface and phylogenic conservation of the Nrf2-Keap1 system. *Genes Cells* 2002;7(8):807-820.
Kozakov, D., *et al.* How good is automated protein docking? *Proteins* 2013;81(12):2159-2166.
Kozakov, D., *et al.* PIPER: an FFT-based protein docking program with pairwise potentials. *Proteins* 2006;65(2):392-406.
Kozakov, D., *et al.* Optimal clustering for detecting near-native conformations in protein docking. *Biophys J* 2005;89(2):867-875.
Ohashi, E., *et al.* Identification of a novel REV1-interacting motif necessary for DNA polymerase kappa function. *Genes Cells* 2009;14(2):101-111.
Olesen, L.E., *et al.* Solitary and repetitive binding motifs for the AP2 complex alpha-appendage in amphiphysin and other accessory proteins. *J Biol Chem* 2008;283(8):5099-5109.
Poy, F., *et al.* Crystal structures of the XLP protein SAP reveal a class of SH2 domains with extended, phosphotyrosine-independent sequence recognition. *Mol Cell* 1999;4(4):555-561.
Rudiger, S., *et al.* Substrate specificity of the DnaK chaperone determined by screening cellulose-bound peptide libraries. *EMBO J* 1997;16(7):1501-1507.
Schuetz, A., *et al.* Structural basis for molecular recognition and presentation of histone H3 by WDR5. *EMBO J* 2006;25(18):4245-4252.
Sheng, Y., *et al.* Molecular recognition of p53 and MDM2 by USP7/HAUSP. *Nat Struct Mol Biol* 2006;13(3):285-291.

Smith, D.M., *et al.* Docking of the proteasomal ATPases' carboxyl termini in the 20S proteasome's alpha ring opens the gate for substrate entry. *Mol Cell* 2007;27(5):731-744.
Srinivasula, S.M., *et al.* A conserved XIAP-interaction motif in caspase-9 and Smac/DIABLO regulates caspase activity and apoptosis. *Nature* 2001;410(6824):112-116.
Ting, D., *et al.* Neighbor-dependent Ramachandran probability distributions of amino acids developed from a hierarchical Dirichlet process model. *PLoS Comput Biol* 2010;6(4):e1000763.
Warbrick, E. PCNA binding through a conserved motif. *Bioessays* 1998;20(3):195-199.
